# Correlation between high blood IL-6 level, hyperglycemia, and glucose control in septic patients

**DOI:** 10.1186/cc11301

**Published:** 2012-04-11

**Authors:** Masataka Nakamura, Shigeto Oda, Tomohito Sadahiro, Eizo Watanabe, Ryuzo Abe, Taka-aki Nakada, Yasumasa Morita, Hiroyuki Hirasawa

**Affiliations:** 1 Department of Emergency and Critical Care Medicine, Graduate School of Medicine, Chiba University, 1-8-1 Inohana Chuo, Chiba-city 2608677, Japan

## Abstract

**Introduction:**

The aim of the present study was to investigate the relationship between the blood IL-6 level, the blood glucose level, and glucose control in septic patients.

**Methods:**

This retrospective observational study in a general ICU of a university hospital included a total of 153 patients with sepsis, severe sepsis, or septic shock who were admitted to the ICU between 2005 and 2010, stayed in the ICU for 7 days or longer, and did not receive steroid therapy prior to or after ICU admission. The severity of stress hyperglycemia, status of glucose control, and correlation between those two factors in these patients were investigated using the blood IL-6 level as an index of hypercytokinemia.

**Results:**

A significant positive correlation between blood IL-6 level and blood glucose level on ICU admission was observed in the overall study population (*n *= 153; *r *= 0.24, *P *= 0.01), and was stronger in the nondiabetic subgroup (*n *= 112; *r *= 0.42, *P *< 0.01). The rate of successful glucose control (blood glucose level < 150 mg/dl maintained for 6 days or longer) decreased with increase in blood IL-6 level on ICU admission (*P *< 0.01). The blood IL-6 level after ICU admission remained significantly higher and the 60-day survival rate was significantly lower in the failed glucose control group than in the successful glucose control group (*P *< 0.01 and *P *< 0.01, respectively).

**Conclusions:**

High blood IL-6 level was correlated with hyperglycemia and with difficulties in glucose control in septic patients. These results suggest the possibility that hypercytokinemia might be involved in the development of hyperglycemia in sepsis, and thereby might affect the success of glucose control.

## Introduction

An elevated blood glucose level in critically ill patients is termed stress hyperglycemia. Several studies have documented that stress hyperglycemia affects outcomes in patients with various clinical conditions requiring intensive care, such as myocardial infarction, cerebrovascular disorders, or traumatic brain injury [1-3]. Hyperglycemia has also been indicated to have deleterious effects on patients with sepsis [4-6]. The Surviving Sepsis Campaign guidelines have therefore consistently recommended blood glucose control with a goal of < 150 mg/dl, since the publication of the original version in 2004 [7-9].

In the pathophysiology of sepsis, proinflammatory cytokines including TNFα, IL-1, and IL-6 are known to play a pivotal role, and overproduced cytokines enter into the bloodstream causing hypercytokinemia, which leads to organ failure via humoral mediator network activation and vascular endothelial damage [10,11].

We have routinely measured blood levels of IL-6 in all patients with sepsis since 2000 to assess the severity of hypercytokinemia, and we have reported the clinical usefulness of the routine measurement of IL-6 levels in septic patients [12,13]. Several other clinical studies have also demonstrated that measurement of the blood IL-6 level is useful as a biomarker of hypercytokinemia [14-16]. We have also implemented blood glucose control in patients with sepsis according to the Surviving Sepsis Campaign guidelines since 2004 [7]. Based on our daily clinical experience in critical care of septic patients, we hypothesize that a correlation exists between the blood IL-6 level and hyperglycemia in sepsis.

With this background, we retrospectively investigated the correlation between blood IL-6 level and blood glucose level in patients with sepsis. We also investigated the relationship between the blood IL-6 level and glucose control in the same patients.

## Materials and methods

### Patients

This retrospective observational study was conducted in patients with sepsis who were admitted to the ICU of Chiba University Hospital (an eight-bed general ICU annually admitting approximately 800 to 1,000 medical/surgical patients, both adult and pediatric) from 2005 to 2010. The inclusion criteria for this study were: age 18 years or older, and diagnosis with sepsis on ICU admission according to the definition of the American College of Chest Physicians/Society of Critical Care Medicine Consensus Conference [17]. The exclusion criteria were: ICU stay shorter than 7 days (too short for sufficient observation of changes in blood glucose level or appropriate determination of effects of glucose control), and steroid therapy during the ICU stay (potentially influencing blood glucose level and inflammatory reaction).

Prior to the initiation of critical care in the ICU, each patient or his/her relative provided written informed consent to possible research use of imaging and blood testing data required for clinical practice as well as information regarding the effectiveness of various treatments, with confidentiality of personal information secured. This retrospective study was approved by the Chiba University Hospital Clinical Research Center Ethics Committee before initiation of the research.

### Patient management for glucose control

We have implemented blood glucose control in all septic patients, adopting the goal of < 150 mg/dl recommended in the Surviving Sepsis Campaign guidelines [7-9]. In addition to an upper-limit blood glucose goal of < 150 mg/dl, a lower-limit blood glucose goal of > 100 mg/dl was adopted to minimize the risk of hypoglycemia. Arterial blood samples for blood glucose monitoring were collected via an intra-arterial pressure monitoring line, and blood glucose was measured with a glucose analyzer built in a blood gas analysis system (Stat Profile pHOx Plus M; NOVA Biomedical Japan, Tokyo, Japan).

Parenteral nutrition was initiated with glucose, amino acids, and vitamins at 100 to 400 kcal/day. Early transition to enteral nutrition was attempted as soon as possible if the cardiopulmonary function of the patient was stable, and unless there was bowel dysfunction in the patient. In patients with septic shock, enteral nutrition was initiated after recovery from shock. Total energy intake was gradually increased by increasing doses of enteral nutrition. Energy intake was also increased with special care to avoid hyperglycemia. To reduce the blood glucose level, regular insulin was continuously administered intravenously using an infusion pump. Measurement of the blood glucose level and adjustment of the infusion rate of intravenous nutritional solution and insulin were all implemented by intensivists only, not by nurses and non-intensivist doctors. The intervals of measurement of blood glucose level and adjustment of infusion rate of intravenous nutrition solution and insulin were basically 3 to 6 hours. However, intensivists shortened measurement intervals, and changed glucose/insulin doses more frequently, if they judged it necessary.

According to the management protocol of the present study, blood glucose control was performed in all patients, aiming to achieve the blood glucose goals within 24 hours after ICU admission. Successful blood glucose control was defined as a blood glucose level of < 150 mg/dl achieved within 24 hours after ICU admission and maintained thereafter within a range between 100 and 150 mg/dl for at least 6 days (that is, up to ICU day 7).

### Blood IL-6 measurement

The blood IL-6 level was determined by chemiluminescent enzyme immunoassay method. A rapid assay system (Human IL-6 CLEIA; Fujirebio, Tokyo, Japan), requiring an assay time of approximately 30 minutes, was used for monitoring the blood IL-6 level with an automated device (Lumipulse f^®^; Fujirebio) [12,13]. The blood IL-6 level thus obtained was used as an index of hypercytokinemia.

### Comparative parameters and statistical analysis

All continuous variables are expressed as the mean ± standard deviation. Normal distributions were confirmed for all continuous variables other than the blood IL-6 level. The blood IL-6 level was used for statistical analysis after logarithmic conversion, since a normal distribution was confirmed for logarithmically converted values of this parameter.

Background parameters were compared between two patient groups, those with successful glucose control and those with failed glucose control, using the unpaired *t *test (continuous variables) and the chi-square test (dichotomous variables). The correlation between blood glucose level and blood IL-6 level on ICU admission (that is, at baseline) was examined by calculating Pearson's product-moment correlation coefficient. The correlations between the blood glucose level and other biomarkers (lactate, C-reactive protein, and so forth) or severity scores, such as the Acute Physiology and Chronic Health Evaluation (APACHE) II or the Sequential Organ Failure Assessment score on ICU admission (that is, at baseline), were also examined by calculating Pearson's product-moment correlation coefficient.

The rate of successful blood glucose control was compared among three patient groups stratified by blood IL-6 level on ICU admission using the chi-square test. Time-course changes in the blood glucose level, the blood IL-6 level, energy intake, and insulin dose during the ICU stay were compared between the successful and failed blood control groups by repeated-measures analysis of variance. For patients in the successful glucose control group, the correlation between insulin dose on ICU day 1 (required for achievement of blood glucose goal < 150 mg/dl) and blood IL-6 level on ICU admission was further examined by calculating Pearson's product-moment correlation coefficient. The 28-day and 60-day survival rates were compared between the successful and failed glucose control groups using the chi-square test. Statistical package software (PASW Statistics 18 for Windows; SPSS Japan Inc., Tokyo, Japan) was used for all statistical analyses, with *P *< 0.05 considered significant.

## Results

### Patients

A total of 255 septic patients were admitted to our ICU during the period from 2005 to 2010: 203 patients stayed for 7 days or longer in the ICU, while 34 and 18 patients were discharged alive and dead, respectively, from the ICU within 7 days and therefore were excluded from this study. After further exclusion of 50 patients having received steroids before or immediately after ICU admission, 153 patients were finally included in this study.

In the overall study population, subjects were aged 64.6 ± 14.2 years (mean ± standard deviation) and male subjects comprised the majority (*n *= 109, 71.2%). The APACHE II score of the total 153 patients was 23.5 ± 7.0 and the Sequential Organ Failure Assessment score of the total 153 patients was 8.2 ± 3.5. Forty-one patients (26.8%) had diabetes mellitus. The 153 patients included 30 with sepsis (19.6%), 86 with severe sepsis (56.2%), and 37 with septic shock (24.2%). Other background parameters are shown in Table 1. The background parameters were also compared between the successful and failed glucose control groups. While the percentage of male patients was significantly higher in the successful glucose control group, no significant difference in age, medical/surgical ratio, or prevalence of diabetes mellitus was observed between the two groups. A significant difference in APACHE II score on ICU admission was observed between the successful and failed glucose control groups (*P *= 0.018). A significant difference in the APACHE II score on ICU admission was observed between the successful and failed glucose control groups (*P *= 0.018). The lactate level and the IL-6 level were also significantly different between the two groups (*P *= 0.035 and *P *< 0.0001, respectively). The percentage of patients with septic shock was significantly higher in the failed glucose control group (*P *< 0.001).

**Table 1:** Patients' background factors

	Overall	Successful glucose control group	Failed glucose control group	*P *value
*n*	153	94	59	
Age (years)	64.6 ± 14.2	65.4 ± 15.1	63.4 ± 12.6	0.394^a^
Gender (male)	109 (71.2%)	74 (78.7%)	35 (59.3%)	< 0.01^b^
Medical/surgical, medical	57 (37.2%)	36 (38.3%)	21 (35.6%)	0.736^b^
Diabetes mellitus	41 (26.8%)	24 (25.5%)	17 (28.8%)	0.656^b^
On ICU admission				
APACHE II score	23.5 ± 7.0	22.5 ± 7.1	25.2 ± 6.6	0.018^a^
SOFA score	8.2 ± 3.5	7.8 ± 3.7	8.7 ± 3.1	0.109^a^
White blood cells (/mm^3^)	14,300 ± 10,300	15,100 ± 10,800	13,000 ± 9,500	0.218^a^
C-reactive protein (mg/dl)	19.5 ± 10.0	18.6 ± 9.8	20.8 ± 10.3	0.203^a^
Lactate (mg/dl)	3.8 ± 2.8	3.4 ± 2.8	4.4 ± 2.8	0.035^a^
IL-6 (pg/ml)	13,800 ± 48,800	5,350 ± 13,800	27,200 ± 75,100	< 0.0001^a^
Type of sepsis				< 0.001^b^
Sepsis	30 (19.6%)	27 (28.7%)	3 (5.1%)	
Severe sepsis	86 (56.2%)	56 (59.6%)	30 (50.8%)	
Septic shock	37 (24.2%)	11 (11.7%)	26 (44.1%)	
Primary source of infection				0.276^b^
Central nervous system	5 (3.3%)	2 (2.1%)	3 (5.1%)	
Neck/mediastinum	19 (12.4%)	11 (11.7%)	8 (13.6%)	
Lung	45 (29.4%)	33 (35.1%)	12 (20.2%)	
Abdomen	47 (30.7%)	25 (26.6%)	22 (37.3%)	
Urinary tract	16 (10.5%)	8 (8.5%)	8 (13.6%)	
Bones/joints/soft tissue	12 (7.8%)	7 (7.4%)	5 (8.5%)	
Others	9 (5.9%)	8 (8.5%)	1 (1.7%)	
Implementation of enteral nutrition within 7 days	89 (58.2%)	55 (58.5%)	34 (58.2%)	0.914^b^
Start of enteral nutrition after ICU admission (days)	3.5 ± 2.1	3.7 ± 2.1	3.3 ± 2.1	0.446

Data presented as mean ± standard deviation or *n *(%). See 'Patient management for glucose control' in Materials and methods for definition of successful glucose control. APACHE, Acute Physiology and Chronic Health Evaluation; SOFA: Sequential Organ Failure Assessment. ^a^Unpaired *t *test. ^b^Chi-square test.

### Correlation between blood glucose level and various parameters on ICU admission

Correlation coefficients (*r*) between the blood glucose level and various parameters (age, APACHE II score, Sequential Organ Failure Assessment score, lactate level, white blood cells, C-reactive protein and IL-6 level) were calculated and are shown in Table 2. In the analysis of the study population, both the APACHE II score and IL-6 level were weakly correlated with the blood glucose level on ICU admission (APACHE II, *r *= 0.25, *P *= 0.01; IL-6 level, *r *= 0.24, *P *= 0.01). When subgroup analysis was performed on diabetic and nondiabetic patients, a more significant positive correlation between blood IL-6 level and blood glucose level on ICU admission was observed in the nondiabetic subgroup (*r *= 0.42, *P *< 0.01).

**Table 2:** Correlation between blood glucose level and various parameters on ICU admission

Glucose level (mg/dl)	Overall (*n *= 153)	Diabetes (*n *= 41)	Nondiabetes (*n *= 112)
vs. age (years)	0.06 (0.47)	0.29 (0.06)	0.02 (0.81)
vs. APACHE II score	0.25 (0.01)	0.28 (0.07)	0.25 (0.01)
vs. SOFA score	0.09 (0.28)	0.09 (0.57)	0.06 (0.56)
vs. lactate (mg/dl)	0.03 (0.79)	0.02 (0.89)	0.08 (0.53)
vs. White blood cells (/mm^3^)	0.01 (0.92)	0.13 (0.40)	0.12 (0.23)
vs. C-reactive protein (mg/dl)	0.14 (0.09)	0.26 (0.10)	0.03 (0.79)
vs. IL-6 (pg/ml)	0.24 (0.01)	0.06 (0.62)	0.42 (< 0.01)

Data expressed as correlation coefficient (*P *value). Pearson's product-moment correlation coefficients were calculated. APACHE, Acute Physiology and Chronic Health Evaluation; SOFA: Sequential Organ Failure Assessment.

### Rate of successful glucose control among three patient groups stratified by blood IL-6 level on ICU admission

**Figure 1:**
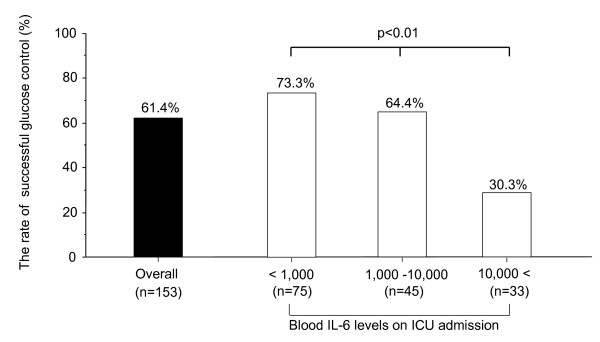
**Rate of successful glucose control among patients stratified by blood IL-6 level at ICU admission**. Comparison of the rate of successful glucose control among three patient groups stratified by blood IL-6 level at ICU admission. Examined by the chi-square test.

Figure 1 shows the rate of successful glucose control calculated for three different patient groups stratified by blood IL-6 level on ICU admission: < 1,000 pg/ml (low), 1,000 to 10,000 pg/ml (medium), and 10,000 pg/ml (high). While the rate of successful glucose control on the overall study population was 61.4% (55/75), the values calculated for the low, medium, and high blood IL-6 level groups were 73.3% (55/75), 64.4% (29/45), and 30.3% (10/38), respectively. A significant difference in the rate of successful glucose control was demonstrated among these three groups (*P *< 0.01, chi-square test).

### Time-course changes in energy intake, insulin dose, blood glucose level, and blood IL-6 level during an ICU stay over 7 days between successful and failed glucose control groups

**Figure 2:**
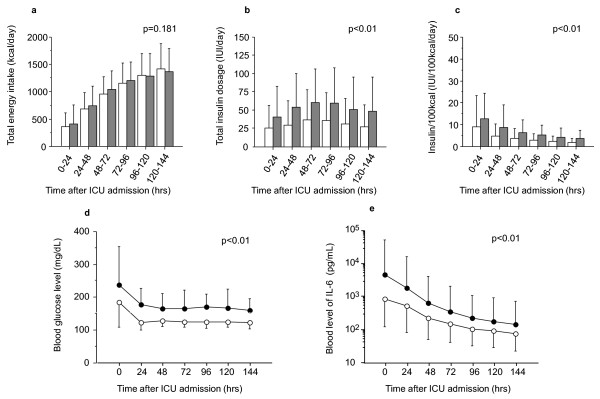
**Time-dependent changes in energy intake, insulin dose, blood glucose level, and blood IL-6 level**. Comparison of time-course changes between the successful and failed glucose control groups: **(a) **total energy intake, **(b) **insulin dose, **(c) **daily insulin dose per 100 kcal energy intake, **(d) **blood glucose level, and **(e) **blood IL-6 level. Open symbols, successful glucose control group; shaded symbols, failed glucose control group. Examined by repeated-measures analysis of variance.

Figure 2 shows a comparison of time-dependent changes in energy intake, insulin dose, blood glucose level, and blood IL-6 level during the first 7 days of ICU stay between the successful and failed glucose control groups. Daily energy intake gradually increased during the first 7 days of the ICU stay, with no significant difference between the two groups (*P *= 0.181; Figure 2a). The daily insulin dose was significantly higher in the failed glucose control group (*P *< 0.01; Figure 2b). The daily insulin dose per 100 kcal energy intake was significantly higher in the failed glucose control group than in the successful glucose control group (*P *< 0.01; Figure 2c). The blood levels of glucose (*P *< 0.01; Figure 2d) and IL-6 (*P *< 0.01; Figure 2e) were significantly higher in the failed glucose control group both on ICU admission and thereafter. In the failed glucose control group compared with the successful glucose control group, the blood glucose level remained higher in spite of a higher dose of insulin, and the blood IL-6 level remained higher.

### Correlation between blood IL-6 level on ICU admission and insulin dose on ICU day 1 in patients in the successful glucose control group

**Figure 3:**
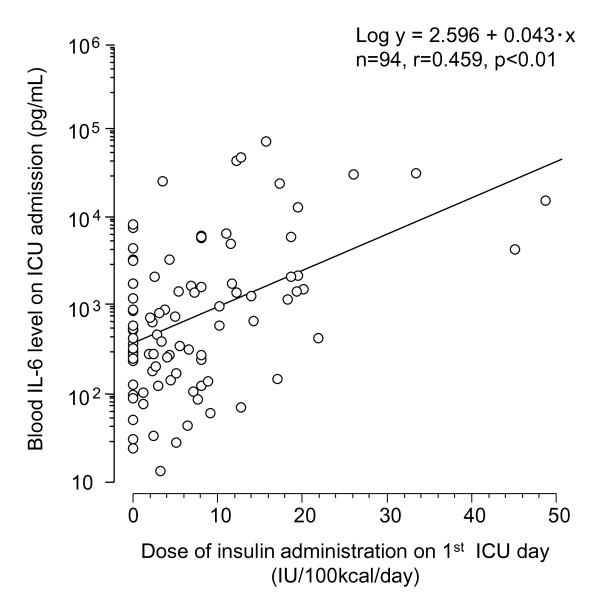
**Correlation between blood IL-6 level on ICU admission and insulin dose on ICU day 1**. Correlation between the blood IL-6 level on ICU admission and the insulin dose on ICU day 1 in patients in the successful glucose control group. Pearson's product moment correlation coefficient was calculated.

Changes in the blood IL-6 level and daily insulin dose were further investigated in the successful glucose control group (*n *= 94). Both the daily insulin dose and the blood IL-6 level were the highest on ICU day 1 and tended to gradually decrease over time thereafter (Figure 2c, e). A significant positive correlation was observed between the values of these two parameters on ICU day 1 (*r *= 0.459, *P *< 0.01; Figure 3).

### Outcomes

Hypoglycemia (glucose level < 60 mg/dl) occurred in four of the 153 patients studied (2.6%), and one of the four hypoglycemic patients died. The 28-day survival rate was 89.5% in the overall study population: the survival rate in the successful glucose control group was significantly higher than that in the failed glucose control group (94.6% vs. 81.4%, *P *< 0.01). The 60-day survival rate in the overall study population was 80.4%, and that in the successful glucose control group and the failed glucose control group was 88.3%, and 67.8%, respectively (*P *< 0.01; Table 3).

**Table 3:** Comparison of 28-day and 60-day survival rates between successful and failed glucose control groups

	Overall	Successful glucose control group	Failed glucose control group	*P *value
*n*	153	94	59	
28-day survivors	137 (89.5)	89 (94.6)	48 (81.4)	< 0.01^a^
60-day survivors	123 (80.4)	83 (88.3)	40 (67.8)	< 0.01^a^

Data presented as *n *(%). See 'Patient management for glucose control' in Materials and methods for definition of successful glucose control. ^a^Chi-square test.

## Discussion

In the present study we investigated the relationship between the IL-6 level, the glucose level, and glucose control in septic patients. The main findings of the present study are as follows. First, a significant correlation was observed between the blood IL-6 level and the blood glucose level on ICU admission (Table 2). Second, the rate of successful glucose control decreased with an increase in the blood IL-6 level on ICU admission (Figure 1). Third, in the failed glucose control group the insulin dose per 100 kcal energy intake was higher (Figure 2c) and glucose control was more difficult (Figure 2d), with the blood IL-6 level remaining higher (Figure 2e). Finally, a significant positive correlation was observed between the blood IL-6 level and daily insulin dose (required for achievement of the blood glucose goal) on ICU day 1 in the successful glucose control group (Figure 3). These results suggest that a high IL-6 level is associated with hyperglycemia and difficulties in glucose control. Furthermore, in the comparison of each parameter on ICU admission between the successful and failed glucose control groups, the APACHE II score, lactate level, and IL-6 level were significantly different. *P *values concerning comparison of the IL-6 level were highest when comparing all parameters (Table 1). In the comparison of correlation coefficients between the blood glucose level and various parameters, the correlation coefficients between IL-6 and glucose levels in nondiabetic patients were the highest of all of the correlation coefficients, as shown in Table 2. From these results, it is suggested that high IL-6 level is an independent marker of severity of sepsis.

Proinflammatory cytokines play a pivotal role in the pathophysiology of sepsis, and overproduced cytokines enter the bloodstream causing hypercytokinemia, which induces various pathophysiological changes and eventually results in organ failure [10,11]. We have measured the blood level of IL-6 for real-time monitoring of the severity of hypercytokinemia, and previously reported the usefulness of blood IL-6 measurements in patients with sepsis [12,13]. Although a limitation has existed in single cytokine measurement, several other studies also demonstrated that the blood level of IL-6 was useful as a biomarker of hypercytokinemia [14-16].

While involvement of stress hormones (for example, corticosteroids, glucagon, catecholamines) in the mechanism of stress hyperglycemia has been repeatedly described, proinflammatory cytokines can cause hyperglycemia by various mechanisms [4-6]. For example, TNFα is known to induce insulin resistance by enhancing secretion of stress hormones [18]. In a rat model of zymosan-induced inflammatory response, Petit and colleague reported that an increase in the blood TNFα level induced a reduction in glucose uptake in skeletal muscle [19]. IL-6 is known to inhibit tyrosine phosphorylation of insulin receptor substrate-1 in isolated human adipocytes, and thereby to suppress glycogen synthesis [20]. In several clinical studies, the serum level of TNFα or IL-6 was increased and glucose tolerance was impaired in diabetics compared with nondiabetics [21,22]. Combining these results of previous basic and clinical research with our findings, these results suggest that hypercytokinemia might be involved in the development of hyperglycemia in sepsis and thereby might affect the success of glucose control.

Although two large-scale clinical studies on glucose control in septic patients previously failed to demonstrate clinical significance of glucose control due to high incidence of hypoglycemic events [23,24], recent review articles suggest that stabilization of the blood glucose level is important in the management of sepsis [4-6]. In our present study, the patients with successful glucose control showed better outcome than the patients with failed glucose control (Table 3). These results from our study also support the importance of glucose control in septic patients.

While the clinical efficacy of glucose control in sepsis has been demonstrated, our clinical experience suggests that achievement of glucose control may remain difficult. Recently, three strategies of successful glucose control have been reported [25,26]. One strategy is statistical process control using a newly developed glucose control protocol on the basis of statistical analysis of previous blood glucose level data [25]. Another is a computer-generated alert system that involves an electronic blood glucose alert in the bedside computer [26]. The third strategy involves the application of an artificial endocrine pancreas, a mechanical glucose control system that is capable of automatic blood sampling at short intervals, measurement of blood glucose levels, and automatic adjustment of insulin and glucose dose [27].

Considering that a high IL-6 level correlated with glucose control in the present study, there is a possibility that improving hypercytokinemia is a new strategy for successful glucose control in addition to above three strategies. Blood purification such as continuous hemodiafiltration is one of the most promising treatments available for the treatment of hypercytokinemia [28,29]. We have reported that continuous hemodiafiltration using a polymethylmethacrylate membrane hemofilter with cytokine-adsorbing capacity successfully improved hypercytokinemia [29]. Although there is little evidence, these blood purifications might be expected to control hypercytokinemia and thus to facilitate glucose control.

The present study has several limitations. First, although we found correlation between a high IL-6 level and hyperglycemia in the present study, it is difficult to elucidate the interaction between high IL-6 level and hyperglycemia. Previous studies suggest that high proinflammatory response might be one of the causes of hyperglycemia. However, additional study is needed to prove these mechanisms. Second, because there was a weak correlation between the APACHE II score and glucose level on ICU admission, we could not completely deny that IL-6-related hyperglycemia might be an epiphenomenon of severe illness. Furthermore, the observed difference in survival rate between the successful and failed glucose control groups does not necessarily demonstrate effectiveness of blood control in sepsis, since there was a substantial difference in severity scores between these two groups. Future studies employing prospective designs may be needed to further elucidate the relationship between hypercytokinemia and stress hyperglycemia and the relationship between hypercytokinemia and glucose control.

## Conclusion

Our retrospective study including 153 patients with sepsis indicated that a high IL-6 level was correlated with hyperglycemia and difficulties in glucose control. These results suggest there is a possibility that hypercytokinemia might be involved in the development of hyperglycemia in sepsis, and that it might thereby affect the success of glucose control.

## Key messages

• In septic patients, a high IL-6 level, which is an index of hypercytokinemia, correlated with hyperglycemia and difficulties in glucose control.

• Hypercytokinemia might therefore be involved in the development of hyperglycemia in septic patients, and might thereby affect the success of glucose control.

## Abbreviations

APACHE: Acute Physiology and Chronic Health Evaluation; IL: interleukin; TNF: tumor necrosis factor.

## Competing interests

The authors declare that they have no competing interests.

## Authors' contributions

MN carried out the data acquisition, secondary database construction, and drafted the manuscript. SO and HH participated in the design of the study, helped to draft the manuscript, and performed the statistical analysis. EW, RA, TN, and YM conceived of the study, participated in its design and coordination, and helped to draft the manuscript. SO and HH helped to draft, read, and approve the final manuscript. All authors approved the final manuscript for publication.
